# Climate and land use shape the water balance and water quality in selected European lakes

**DOI:** 10.1038/s41598-024-58401-3

**Published:** 2024-04-05

**Authors:** Ma. Cristina Paule-Mercado, Rubén Rabaneda-Bueno, Petr Porcal, Marek Kopacek, Frederic Huneau, Yuliya Vystavna

**Affiliations:** 1 Biology Centre, Institute of Hydrobiology, Academy of Sciences of the Czech Republic, Na Sádkách 7, 37005 České Budějovice, Czech Republic; 2 Faculty of Science, University of South Bohemia in České Budějovice, Branišovská 1760, 370 05 České Budějovice, Czech Republic; 3 Département d’Hydrogéologie, Université de Corse Pascal Paoli, BP52, 20250 Corte, France; 4 Centre National de la Recherche Scientifique (CNRS), UMR 6134 SPE, 20250 Corte, France

**Keywords:** European lakes, Isotope-based modelling, Artificial Intelligence, Climate change, Water quality, Environmental sciences, Hydrology

## Abstract

This study provides insights into factors that influence the water balance of selected European lakes, mainly in Central Europe, and their implications for water quality. An analysis of isotopic, chemical and land use data using statistical and artificial intelligence models showed that climate, particularly air temperature and precipitation, played a key role in intensifying evaporation losses from the lakes. Water balance was also affected by catchment factors, notably groundwater table depth. The study shows that lakes at lower altitudes with shallow depths and catchments dominated by urban or crop cover were more sensitive to water balance changes. These lakes had higher evaporation-to-inflow ratios and increased concentrations of total nitrogen in the water. On the other hand, lakes at higher elevations with deeper depths and prevailing forest cover in the catchment were less sensitive to water balance changes. These lakes, which are often of glacial origin, were characterized by lower evaporation losses and thus better water quality in terms of total nitrogen concentrations. Understanding connections between water balance and water quality is crucial for effective lake management and the preservation of freshwater ecosystems.

## Introduction

Water scarcity is a growing problem worldwide, as freshwater resources are often insufficient to meet human and ecological needs^1–3^. This is due to factors such as water withdrawals, land use change, climate change, and declining groundwater levels^4,5^. As a result, reduced water availability and poor water quality have raised concerns among water users^3,6,7^. The problem is exacerbated in areas where water resources cannot compensate for losses^3^. However, detecting water scarcity hotspots is challenging due to the complex interplay of hydrological, climatic, and socioeconomic factors. Therefore, an approach that analyses indicators of water scarcity across areas and include ecological responses and geographic variability are needed^3,8^.

Lakes adapt to climatic, hydrological and land use changes^8,9^, and integrate multiple changes in the water balance and quality in the catchment^8,10,11^. The ability of lakes to reflect the complex interactions between water balance, water quality, and environmental changes makes them valuable models for understanding and managing water scarcity in a broader catchment context^8,12,13^. However, it has been challenging to compare climate and hydroclimate changes in lakes due to the wide variations in lake characteristics such as size, depth, surrounding land use, and whether they are naturally formed or artificial reservoirs. The use of stable isotopes in the water molecule (^18^O and ^2^H) can provide a solution to this problem. Isotopes that integrate climate and hydrological settings provide a comprehensive record of changes in the catchment and serve as a proxy for understanding the hydrological history of the lake^8,14^.

The initial isotopic composition of water in lakes (a given ratio of heavy and light water isotopes) is determined by various factors such as climate, landscape, and the lake's own characteristics. When water evaporates from a lake, the lighter isotopes are lost to the atmosphere in greater proportions, leading to the enrichment of heavy isotopes in the remaining lake water. This process has been observed and studied by researchers^8,14^ and is well documented in the scientific literature^15,16^. Water balance models are used to constrain evaporation losses and inflow (E/I) and estimate transpiration fluxes in evapotranspiration (T/ET) employing the baseline regional isotope ratio^15,17,18^. The E/I ratio reflects the capacity of a lake and its catchment to compensate water losses by inflow and sustain the catchment water budget that can be used as a proxy of the water availability^8^. T/ET values show the influence of vegetation on the overall water loss from a particular ecosystem or area^18^.

We assume that E/I and T/ET, which reflect the hydrological conditions in the lake catchments, are closely connected to the lake water quality. The effects of changes in lake water balances on nutrient concentrations are widely recognized. For instance, an increase in nutrient concentrations within a lake can lead to eutrophication, which is characterized by the excessive growth of algae and other aquatic plants. On the other hand, a decrease in nutrient concentrations can limit the availability of nutrients to aquatic organisms, leading to reduced productivity in a lake. This understanding highlights the critical relationship between water balance and nutrient dynamics, and emphasizes the importance of maintaining a balanced and healthy ecosystem in lakes^19^.

In recent years, the use of machine learning algorithms with open data sources such as HydroLakes, GLEON, WorldClim2, GLDAS, the Noah Land Surface Model, and GEM databases, has emerged as a valuable approach for studying and understanding the factors that drive hydrological processes^20^. Associated tools such as the Random Forest (RF) method have the advantage of being able to identify and analyse complex relationships between variables that often exhibit polynomial patterns^8,21^. The use of these advanced tools and datasets opens an opportunity to gain deeper insights into the complex dynamics of water availability in lakes and catchments. This was the overall goal of this study, with the following main objectives: (i) to estimate the isotopic water balance in 73 European lakes that are mainly in Central Europe; (ii) to develop an artificial intelligence model to understand the dominant factors among climate and catchment parameters that control lake water balances, and (iii) to determine the relationship between isotope-based water balances and nutrients in the lake water.

## Results

### Stable isotope variations and isotope-based water balances in lakes

**Figure 1:**
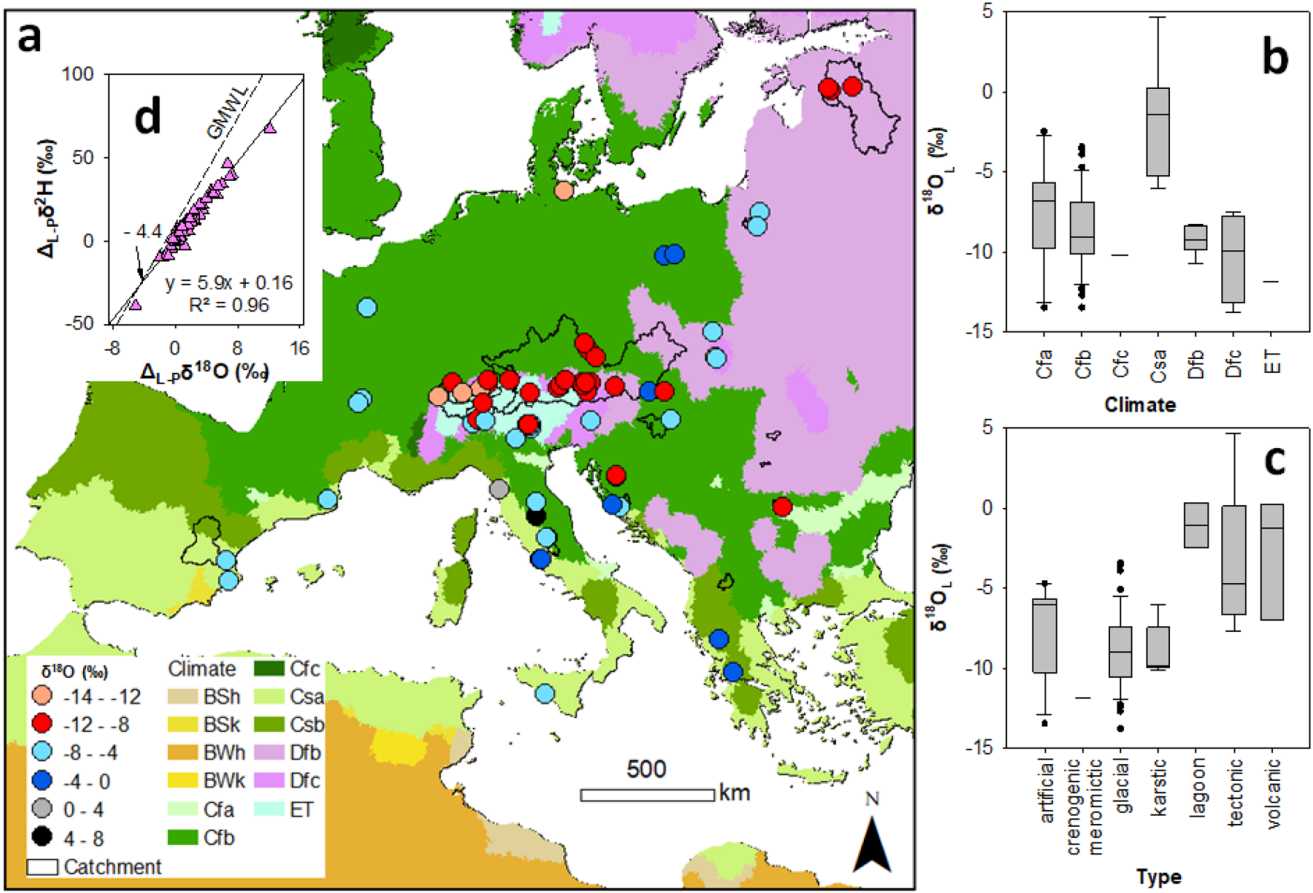
Stable isotope variations in selected European natural and artificial lakes: (**a**) spatial, (**b**) climate type, (**c**) lake type variations of *δ*^18^O_L_ values, and (**d**) the lake-formed local evaporation line (the figure for *δ*^2^H_L_ values is in Fig. S1). The country-level map, taken from HydroBasin^22^, underwent climate classification according to the Köppen–Geiger^23^ climate method before being processed in ArcGIS 10.6.1^24^.

A total of 73 lakes of different types (lagoon, volcanic, tectonic, artificial (water reservoirs), glacial and crenogenic monomictic) were included in this study. The lakes surface areas ranged from 0.004 to 3555 km^2^ and the average depth varied from 1 to 177.4 m. The study included lowland (from − 3.5 m asl: Lake Vrana, Croatia) to high elevation alpine lakes (to 2157 m asl: Vysne Wahlenbergovo, Slovakia) (see Data availability). The selected lakes studied were mainly located in the Mediterranean (C) and cold temperate (D) climate zones (Fig. 1a). The variation in the isotopic composition (*δ*^18^O_L_) of the studied lakes ranged from − 13.8 to 4.7‰, with the most enriched values in lakes located in the hot-summer Mediterranean climate type (Csa) (Fig. 1a,b). Lagoon, tectonic, and volcanic lakes had the highest isotope levels, while artificial and glacial had the most depleted values (Fig. 1c). The isotopic composition of studied lakes formed a local (European) evaporation line (LEL) with the intersection with the Global Meteoric Water Line (GMWL) at − 4.4‰ for *δ*^18^O (Fig. 1d). The slope of the LEL is 5.9, which is less than the slope of the GMWL.

**Figure 2:**
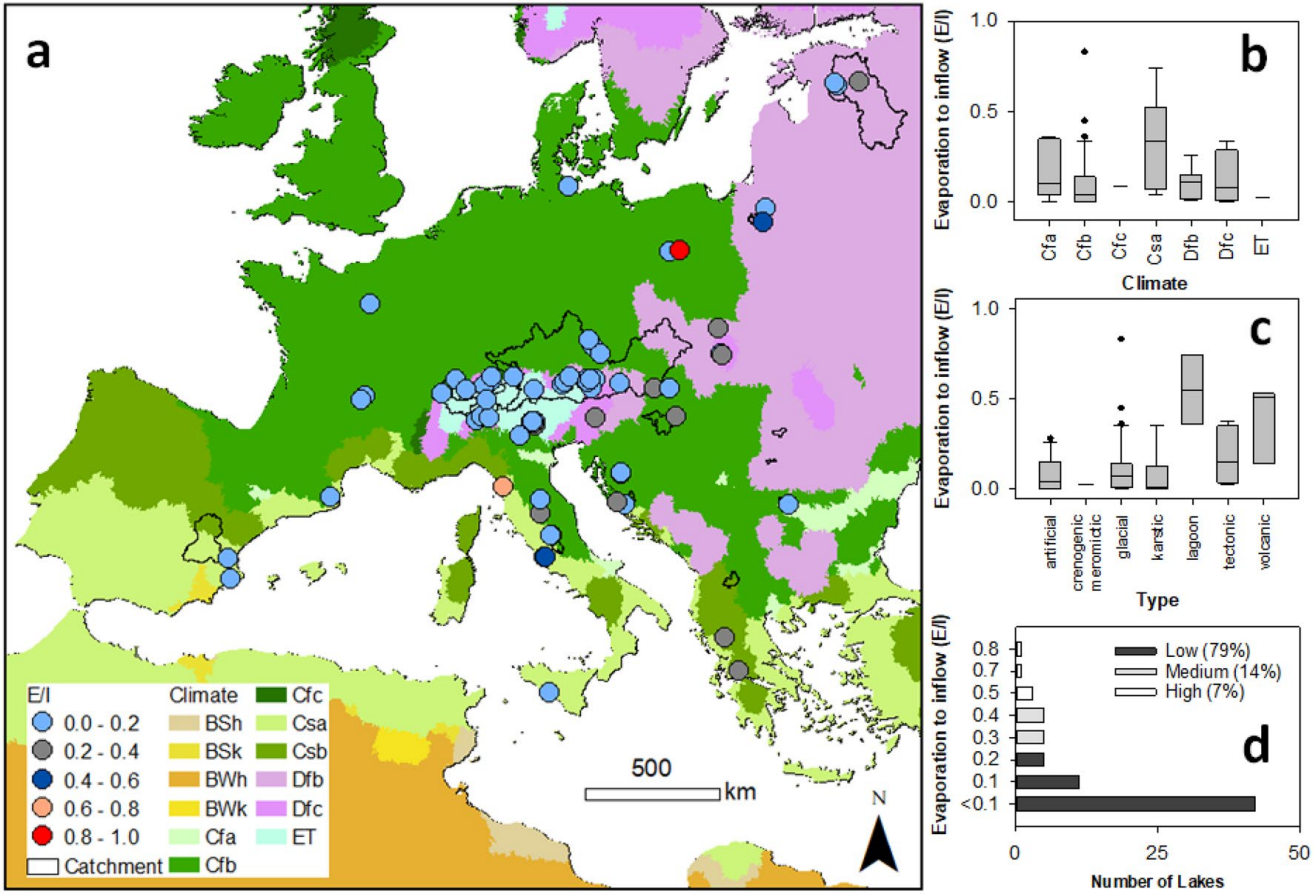
Evaporation to inflow (E/I) according to (**a**) spatial distribution, (**b**) climate, (**c**) types, and (**d**) statistics. The maps, taken from HydroBasin^22^, underwent climate classification according to the Köppen–Geiger^23^ climate method before being processed in ArcGIS 10.6.1^24^.

The estimated isotopic water balance of the lakes showed that most lakes had evaporation to inflow (E/I) rates < 20% (Fig. 2a) with the lowest E/I in cold (ET) climate (Fig. 2b). Only one lagoon and two volcanic lakes had E/I > 40% (Fig. 2c). In total, 21% of studied lakes had medium and high E/I (Fig. 2d).

**Figure 3:**
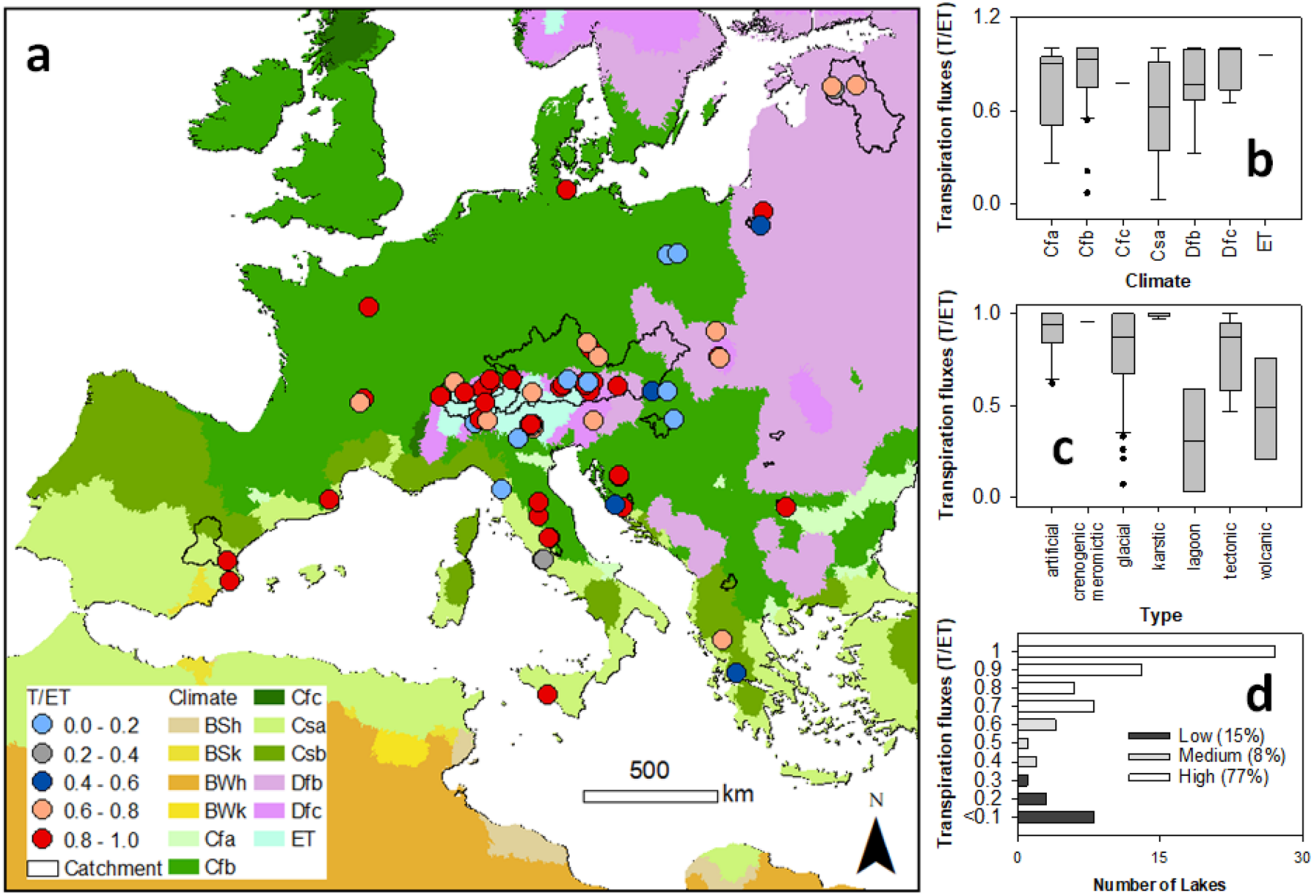
Transpiration to evapotranspiration (T/ET) according to (**a**) spatial distribution, (**b**) climate, (**c**) types, and (**d**) statistics. The maps, taken from HydroBasin^22^, underwent climate classification according to the Köppen–Geiger^23^ climate method before being processed in ArcGIS 10.6.1^24^.

Estimations of T/ET showed that in most lakes (77%) catchment transpiration dominates the evapotranspiration fluxes (Fig. 3a). The lowest T/ET values (< 0.3) were in the catchments of lagoon and volcanic lakes and lakes located in the Csa climate zone (Fig. 3b–d).

**Figure 4:**
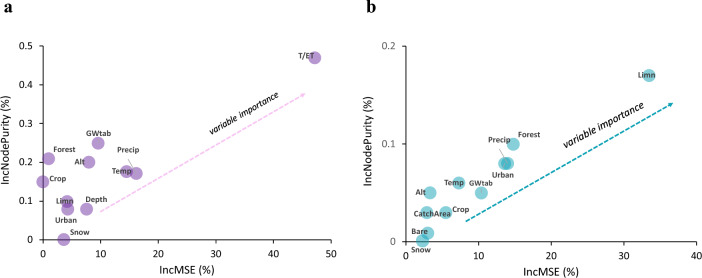
Multi-way importance plots show the importance of individual variables in relation to E/I (**a**) and T/ET (**b**), and are based on the percentage in Mean Squared Error Increase (IncMSE, %) and Mean Decrease Gini (IncNodePurity, %). *Temp* average annual air temperature (°C), *Precip* average monthly precipitation amount (mm), *depth* average lake depth (m), *Snow* snow extent in the catchment (%), *Alt* altitude (m asl), *CatchArea* catchment area (km^2^), *Bare* extent of bare land (%), *Crop* extent of crops (%), *Forest* extent of forests (%), *Urban* extent of urban areas (%), *GWtab* groundwater table depth (cm), *Limn* limnicity (%).

The RF model showed that the E/I rate in lakes was mainly controlled by T/ET, elevation, climate (average temperature and precipitation) and groundwater table depth (with Mean Squared Error Increase (IncMSE) of more than 10%) (Fig. 4a). The model explained 43.6% of the variability (*p* < 0.01). While forest cover in the catchment had a lower inMSE value, the IncNodePurity was higher than for altitude and climate variables, indicating the importance of these two variables in the control of E/I. The RF for T/ET showed that limnicity was the dominant factor controlling T/ET (> 30% of IncMSE) followed by the cover of forest and urban areas, average precipitation and groundwater table depth in the catchment (Fig. 4b). The model explained 39.9% of the variability (*p* < 0.01).

The E/I had significant positive relationships with average temperature, limnicity, cropland and urban area cover, and significant negative relationships with altitude, groundwater table depth, average depth of the lake, average precipitation, bare land and forest cover in the catchment. In contrast, the T/ET had significant positive correlations with altitude, average precipitation amount and forest cover (Table 1). E/I and T/ET showed a significant negative correlation to each other (*p* < 0.01), indicating the highest E/I rate was observed in lakes with the lowest transpiration component in the evapotranspiration (T/ET).

**Table 1:** Pearson correlation between isotopic water balance values (E/T and T/ET), climate and catchment variables.

Primary	Alt	Temp	Precip	GWtab	Depth	Forest	Crop	Bare	Urban	Limn
E/I	**− 0.30**^a,^*	0.36**	− 0.44**	− 0.42**	− 0.26*	− 0.46**	0.35**	− 0.24*	0.29*	0.35**
T/ET	0.23*	**− **0.13	0.26*	0.21	0.01	0.32**	**− **0.20	0.23	**− **0.17	**− **0.22

^a^Numbers are *r*, the Pearson correlation criteria, negative sign (−) indicates negative correlation, asterisks indicate significance: *p < 0.05, **p < 0.01, significant values are in bold. For abbreviations see Fig. 4.

### Relationships between water chemistry, isotope composition and water balance

**Figure 5:**
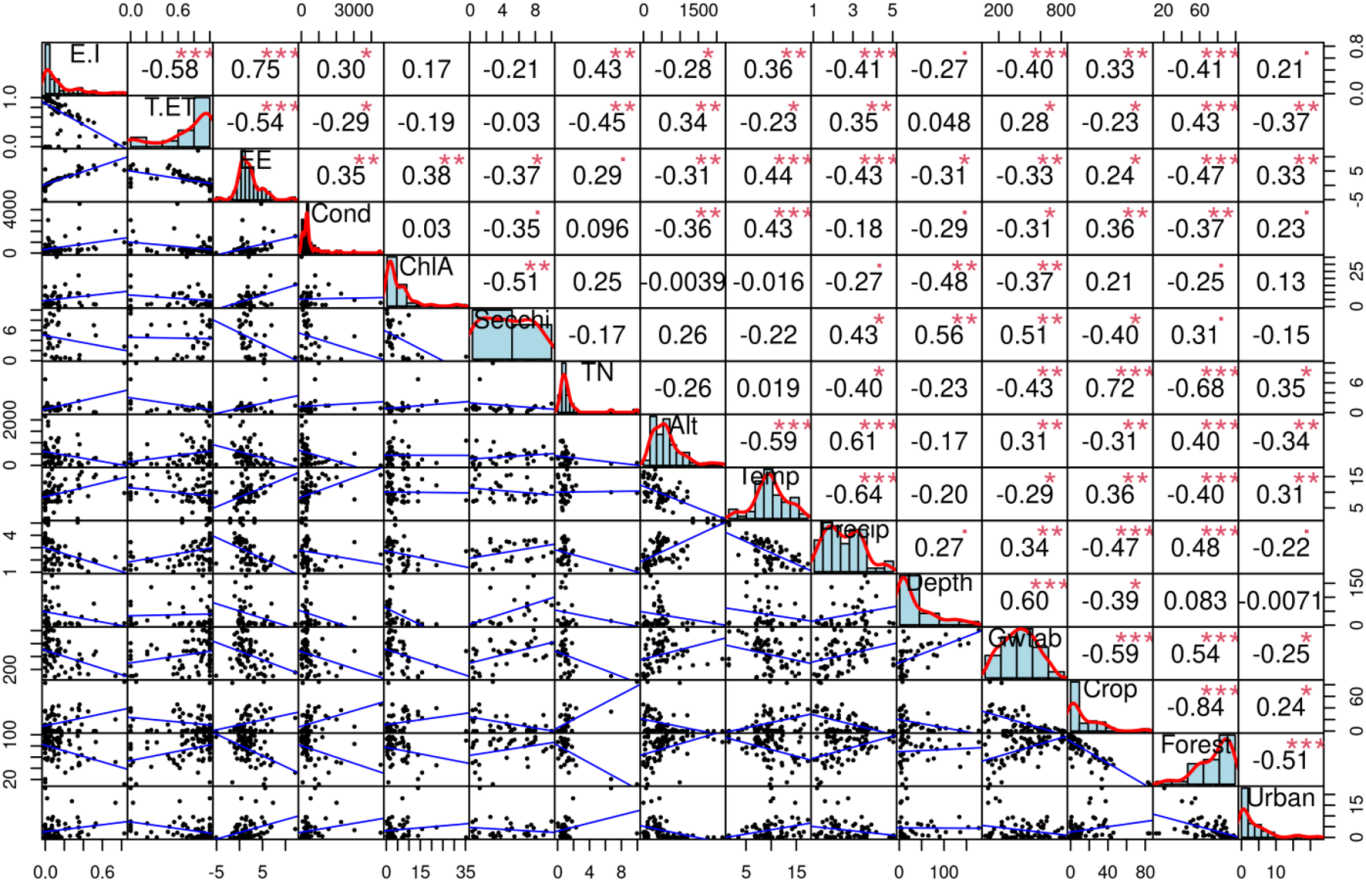
Correlation matrix between variables. Significance levels are shown by the numbers of asterisks: p-values < 0.001 (***), 0.01 (**), 0.05 (*). For abbreviations see Fig. 4.

The correlation analysis showed that E/I had positive correlations (p < 0.05) with total nitrogen (TN), temperature, precipitation, altitude, groundwater table depth, forest and cropland. T/ET had a significant negative correlation with TN, conductivity, cropland and urban cover but significant positive with altitude, precipitation, groundwater table depth and forest cover. Evaporative enrichment (EE), estimated as the difference between isotope values in lake water and catchment-weighted isotope values in precipitation, had significant (p < 0.01) positive correlations with altitude, temperature, conductivity and chlorophyll A, cropland and urban cover, but negative with Secchi depth, which is a measure of the water transparency and with precipitation, groundwater table depth and forest cover (Fig. 5). E/I had positive and negative correlations with NO_2_ and NO_3_^−^ (p < 0.05), respectively. T/ET had a negative correlation with TP (p < 0.05). There were no significant correlations of isotopic parameters (E/I, T/ET and EE) with orthophosphate, total ammonium, dissolved oxygen or pH (Fig. S2).

The parameters correlated with the isotopic parameters reflected the diversity of correlation patterns between lake and catchment characteristics (Fig. 5). In particularly, conductivity had significant positive correlations with average temperature and cropland cover, and significant negative correlations with altitude, groundwater table depth and forest cover in the catchment. Chlorophyll A had significant negative correlations with Secchi depth, average lake depth and groundwater table depth. Secchi depth showed significant positive correlations with average precipitation, average lake depth, and groundwater table depth, and a negative correlation with the cropland area in the catchment (Fig. 5). TN showed significant positive correlations with cropland and urban land covers, and negative correlations with forest cover and groundwater table depth in the catchment.

## Discussion

Insights into the isotope-based water balances of selected European lakes revealed that a minority, less than 10%, exhibited high E/I ratios exceeding 40%, while over 20% had intermediate E/I rates ranging from 20 to 40%. These rates were found to be influenced by a combination of climate and catchment parameters. The influence of climate on lake water balance has been extensively studied and established, with air temperature and precipitation playing critical roles. Specifically, higher temperatures and reduced precipitation have been identified as key climatic factors intensifying evaporation losses from lakes, but increased water withdrawals for socio-economic needs (agriculture, industrial and public water supply, etc.) are also important^25–27^. The strong influence of climate variables such as air temperature and precipitation were also confirmed by the results of the RF model and correlation analysis in our study. In addition to climate, the impacts of catchment parameters such as groundwater and land use on water balance were also found to be significant (Fig. 4). Among the various variables studied, groundwater table depth emerged as the most influential factor affecting lake water balances. This is consistent with other studies on the role of groundwater, which is also related to the site-specific and geographical structures of lake environments, in sustaining the water balance of lakes in Europe^28,29^, including studies where isotope hydrology was applied^30,31^.

We found a negative correlation between groundwater table depth and E/I ratios (Table 1), with shallow aquifers being less effective in compensating for evaporation fluxes. This is due to the shallow groundwater, which depending on the site-specific nature of the soil or substratum can be affected by almost direct evaporation during soil infiltration through the first one or two meters of the ground (in addition to vegetation transpiration/adsorption from roots)^29,32^. Our observations also help explain the phenomenon whereby deeper lakes possess a greater capacity to offset evaporation losses through deeper aquifers that are hydrologically connected to the lakes. In Europe, deeper lakes are often connected to deeper groundwaters (> 100 m) and even confined or semi-confined aquifers usually of larger extent, which are able to provide vast amounts of water to lakes, while shallow lakes are usually only in equilibrium with shallow unconfined aquifers (< 2 m) that are often very close to the surface and affected by evaporation and transpiration^33^. While no significant linear correlation was found between T/ET and groundwater table depth (Table 1), the RF model showed that groundwater table depth strongly influenced T/ET (Fig. 4). This can be explained by that fact that shallow groundwater is more readily available for vegetation and can increase transpiration patterns^34^, which is also supported by the significant positive correlation between T/ET and the extent of forest cover in the catchment (Table 1).

There was a significant negative correlation between E/I and T/ET, indicating that catchments with lower transpiration fluxes relative to evapotranspiration experience greater evaporation losses. These catchments are predominantly characterized by a higher extent of urban areas (Fig. 4b) and can generally be found at lower altitudes (Table 1). Catchments with higher limnicity can have higher evaporation to inflow losses, which can be explained by the longer residence time of water in the catchment and thus higher possible impact by evaporation^8^. Considering that E/I serve as an indicator of a lake's ability to maintain its water balance, lakes at lower altitudes with shallow depths and with catchments dominated by urban or crop cover are most susceptible to changes in the water balance. Conversely, lakes with higher altitudes, deeper depths, and a greater proportion of forest cover in the catchment are less sensitive to variations in the water balance. These lakes are primarily of glacial origin.

Our results revealed a strong connection between the water balance of lakes and their water quality, specifically in terms of TN concentrations, with lakes having higher E/I ratios exhibiting higher TN concentrations. The correlation analysis further indicated that TN concentration levels were related to both crop cover and urban land cover in the catchment, with crop cover having higher significance. The primary source of TN in areas with high crop cover is typically from agricultural practices^35^. Nitrogen is an essential nutrient for plant growth and is commonly supplied to crops through the application of nitrogen-based fertilizers. Synthetic fertilizers that contain nitrogen compounds, such as ammonium nitrate, urea, or ammonium sulphate enhance crop productivity. In addition to fertilizers, other sources of TN in crop cover areas can include organic matter decomposition, such as the breakdown of crop residues or organic amendments like manure or compost. Nitrogen can also be derived from atmospheric deposition, where nitrogen-containing compounds from the air (such as ammonia or nitrogen oxides) are deposited in the catchment through rainfall or dry deposition^36^. Higher TN concentrations in urban areas come from vehicle emissions, industrial activities, wastewater treatment plants, sewage systems, the use of nitrogen-based fertilizers, and atmospheric deposition^35,37^. The specific sources and contributions of TN vary based on factors like population density, industrial activities, transportation patterns, and land use practices^35^.

Our study was biased to lakes mainly located in Central Europe and attributed to cold and Mediterranean climates. But these results provide a basic understanding on the connections between water balance and water quality that is crucial for effective lake management and the preservation of freshwater ecosystems. This research gives a basic information on interaction between climate, catchment, water balance and water quality that can be further used for water resource management. By focusing on high E/I ratios and elevated levels of nitrogen compounds, efforts can be made to protect lakes and their surrounding catchments from anthropogenic impacts and ensure the sustainability of these valuable freshwater resources.

## Conclusion

Climate change can have significant impacts on the water balance of lakes, affecting both the water quantity and quality. By studying the water balance in lakes using stable water molecule isotopes, we can identify potential risks and develop strategies to adapt to these changes. Particularly, since decreases in precipitation have been predicted, we can plan for potential water shortages in the future and implement measures, such as control the land use, to conserve water resources. Additionally, the study of water balances helps us to understand the factors influencing the eutrophication of lakes. By identifying the sources of excessive nutrients in the catchment, such as agricultural runoff or wastewater discharges, we can implement measures to reduce nutrient inputs and prevent such eutrophication. Overall, studying the water balance of lakes provides valuable insights into the functioning of these aquatic systems and allows us to develop sustainable management strategies to protect and preserve their ecosystems. Future studies should focus on the development of climate and land use scenarios that can be applied to water management.

## Methods

The data on water isotopes and water chemistry are comprised of unpublished data from the Institute of Hydrobiology Biology Centre of the Czech Academy of Sciences, International Atomic Energy Agency (IAEA) and Global Network of Isotopes in Rivers (GNIR), as well as from the scientific literature. The catchment polygons were delineated using ArCGIS 10.6.1 and a hydrologically conditioned raster digital elevation mode (DEM) of data from HydroSHEDS^38^; see the “Methods” online. For further information on the data sources and the treatment of environmental data in the Köppen-Geiger climate classification, RCWIP2, Copernicus climate and land cover data, Climate Engine, and HydroATLAS (2019), readers are referred to the “Methods” online. All lake isotope data (δ^18^O and δ^2^H) were normalized to the isotopic composition of each lake catchment’s precipitation amount-weighted inputs^39^, which was defined as the difference between lake isotope values (δ^18^O_L_ or δ^2^H_L_) and precipitation isotopes values (δ^18^O_P_ or δ^2^H_P_) falling on the catchment (Eq. 1):

1 $$\Delta_{L - P} \delta^{18} O\left( {\Delta_{L - P} \delta^{2} H} \right) = \delta^{18} O_{L} \left( {\delta^{2} H_{L} } \right) - \delta^{18} O_{P} \left( {\delta^{2} H_{P} } \right)$$

Evaporative enrichment (EE) values were based on normalized data (_ΔL-P_δ^18^O and Δ_L-P_δ^2^H)^40^ and were converted to median values per lake for lakes with numerous data points. The isotopic equilibrium separation^16^ and isotopic composition of the evaporation flux^15,41^ were utilized to estimate the evaporation to inflow ratios (E/I) using non-normalized lake isotope data. We determined the evaporation/input (E/I) ratios for a subset of 73 lakes. When the isotope-enabled model showed a high discrepancy in the E/I ratio between the O and H two isotopes (i.e., > 20% discrepancy), the lake was considered isotopically unbalanced^8^. Additionally, some lakes could not be considered because only δ^18^O was available for the water balance calculations, or the model could not be applied correctly because of assumed additional water inputs from deep groundwater recharge or glacier melts. Three lakes groups were delimited according to their E/I value: (i) ˂ 0.2 (lakes with low evaporation losses); (ii) 0.2–0.4 (lakes with moderate evaporation losses) and (iii) ˃ 0.4 (lakes with high evaporation losses)^8^. The fraction of the contribution of transpiration to the evapotranspiration fluxes (T/ET, dimensionless value) was estimated based on a simple steady‐state representation of the catchment water balance, similar to previous studies^12,16,37,38^, as Eq. (2):

2 $$T/ET= \frac{P-Q-E/I.P}{P-Q}$$

A Gaussian mixture model based on expectation maximization (EM) and Bayesian information criterion (BIC) was used to cluster the lakes according to their determined T/ET rate (XLSTAT Basic +, v.2021.2). According to the T/ET value, the model findings identified three lake groups: (i) ˂ 0.3 (lakes with low *T/ET*); (ii) 0.3–0.6 (moderate *T/ET*) and (iii) ˃ 0.6 (high *T/ET*).

The local evaporation line (LEL) slopes and intercept of the lakes (the 95% confidence level with a *p*-value < 0.05) were statistically significant, as determined by regression coefficient^44^. We used Random Forest models (R package ‘randomForest’^44,45^) with bootstrapped aggregating to estimate Δ_L-P_δ^18^O and Δ_L-P_δ^2^H as a function of various predictors (Table S1). Out-of-bag (OOB) and mean square error (MSE in %) were applied to estimate model error. The MSE test determined the significance of each predictor variable, with high MSE indicating importance in modeling the lake δ value, and low MSE indicating unimportance.

The residual sum of squares (R^2^)^44^ was used to quantify the model’s capacity to capture the percentage of total variability. The “Methods” online provide a detailed description of the methods, data availability, and references used in this study.

### Supplementary Information

Supplementary Information.

## Data Availability

The datasets used and analysed during the current study available from the corresponding author on reasonable request.
