# Supplementary material for: Climate and land use shape the water balance and water quality in selected European lakes
Source: Sci Rep. 2024 Apr 5;14:8049. doi: 10.1038/s41598-024-58401-3 (PMC10997787; doi:10.1038/s41598-024-58401-3)
Supplement: Supplementary file 1 — Supplementary Information. [file 41598_2024_58401_MOESM1_ESM.docx]

**Supplementary information**

**Climate and land use shape the water balance and water quality in selected European lakes**

Ma. Cristina Paule-Mercado^1^, Rubén Rabaneda-Bueno^1^, Petr Porcal^1^, Marek Kopacek^1^, Frederic Huneau^2,3^, Yuliya Vystavna^1^

^1^ Biology Centre, Academy of Sciences of the Czech Republic, Institute of Hydrobiology, Na Sádkách 7, 37005 České Budějovice, Czech Republic

^2^ Université de Corse Pascal Paoli, Département d'Hydrogéologie, Campus Grimaldi, BP52, 20250 Corte, France

^3^ Centre National de la Recherche Scientifique (CNRS), UMR 6134 SPE, 20250 Corte, France

**Contents**

[1. Supplementary Figures 2](#_Toc160090726)

[**Figure S1. Stable isotope variations in selected European lakes and reservoirs: (a) spatial, (b) Köppen-Geiger climate type, (c) lake type variations of *δ*^2^H_L_ values, and (d) lakes formed local evaporation line.** 2](#_Toc160090727)

[**Figure S2. Correlation matrix between variables. The areas of the circles show the absolute value of the corresponding correlation coefficient. E/I: evaporation to inflow; T/ET: transpiration fluxes; EE: evaporative enrichment; NO_3_^-^: nitrate (mg l^-1^); NO_2_^-^: nitrite (mg l^-1^); PO_4_^3-^ : orthophosphate (mg l^-1^); NH_4_^+^: total ammonium (mg l^-1^); TP: total phosphorus (mg l^-1^); DO: dissolved oxygen (%); and pH. Each significance level is associated to a symbol: *p*-values 0.001 (***), 0.01 (**), 0.05 (*).** 3](#_Toc160090728)

[2. Supplementary Methods 3](#_Toc160090729)

[Water quality and stable isotopes analyses 3](#_Toc160090730)

[Data processing 4](#_Toc160090731)

[Data treatment 5](#_Toc160090732)

[Isotope-enabled modeling 5](#_Toc160090733)

[Random Forest Model 7](#_Toc160090734)

[**Table S1. Primary and the explanatory values (EV) used in the Random Forest model** 8](#_Toc160090735)

[3. References 9](#_Toc160090736)

1. Supplementary Figures

The δ^2^H_L_ values (70 lakes) exhibited similar spatial patterns in European lakes as the δ^18^O_L_ values (73 lakes), although having a smaller dataset. This similarity was seen with regard to the Köppen-Geiger climatic type^1^, as shown in Figure S1.


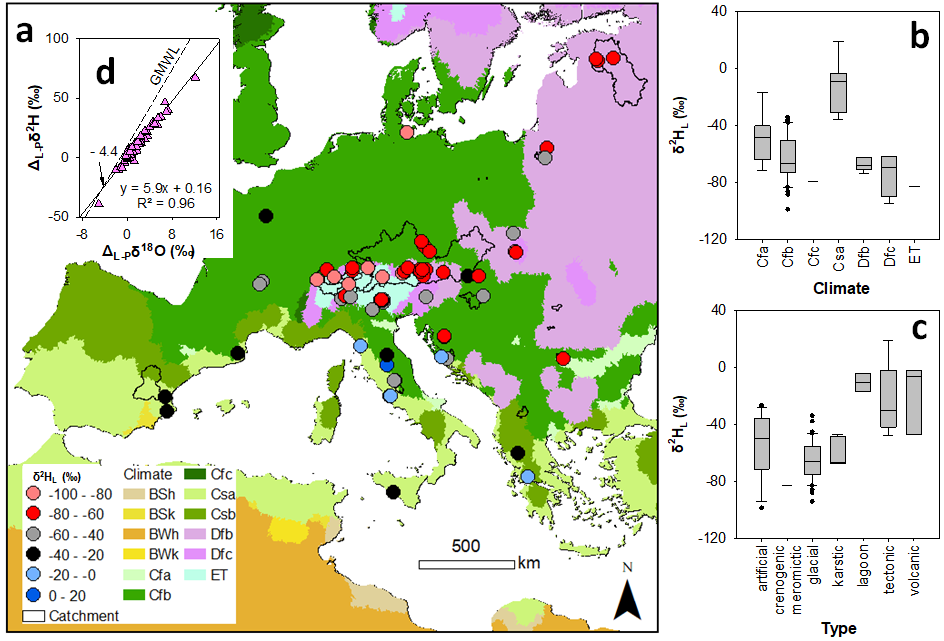


## **Figure S1. Stable isotope variations in selected European lakes and reservoirs: (a) spatial, (b) Köppen-Geiger climate type, (c) lake type variations of *δ*^2^H_L_ values, and (d) lakes formed local evaporation line. The maps, taken from HydroBasin**^2^**, underwent climate classification according to the Köppen-Geiger**^1^ **climate method before being processed in ArcGIS 10.6.1**^3^**.**

E/I had positive and negative correlation with NO_2_ and NO_3_^-^ (*p* < 0.05), respectively. There is a significant positive association (p < 0.05) between T/ET and EE, and a negative correlation with TP. Weak positive correlation (*p* < 0.05) or no significant correlation of isotopic parameters (E/I, T/ET and EE) with orthophosphate, total ammonium, dissolved oxygen and pH was found (Figure S2).


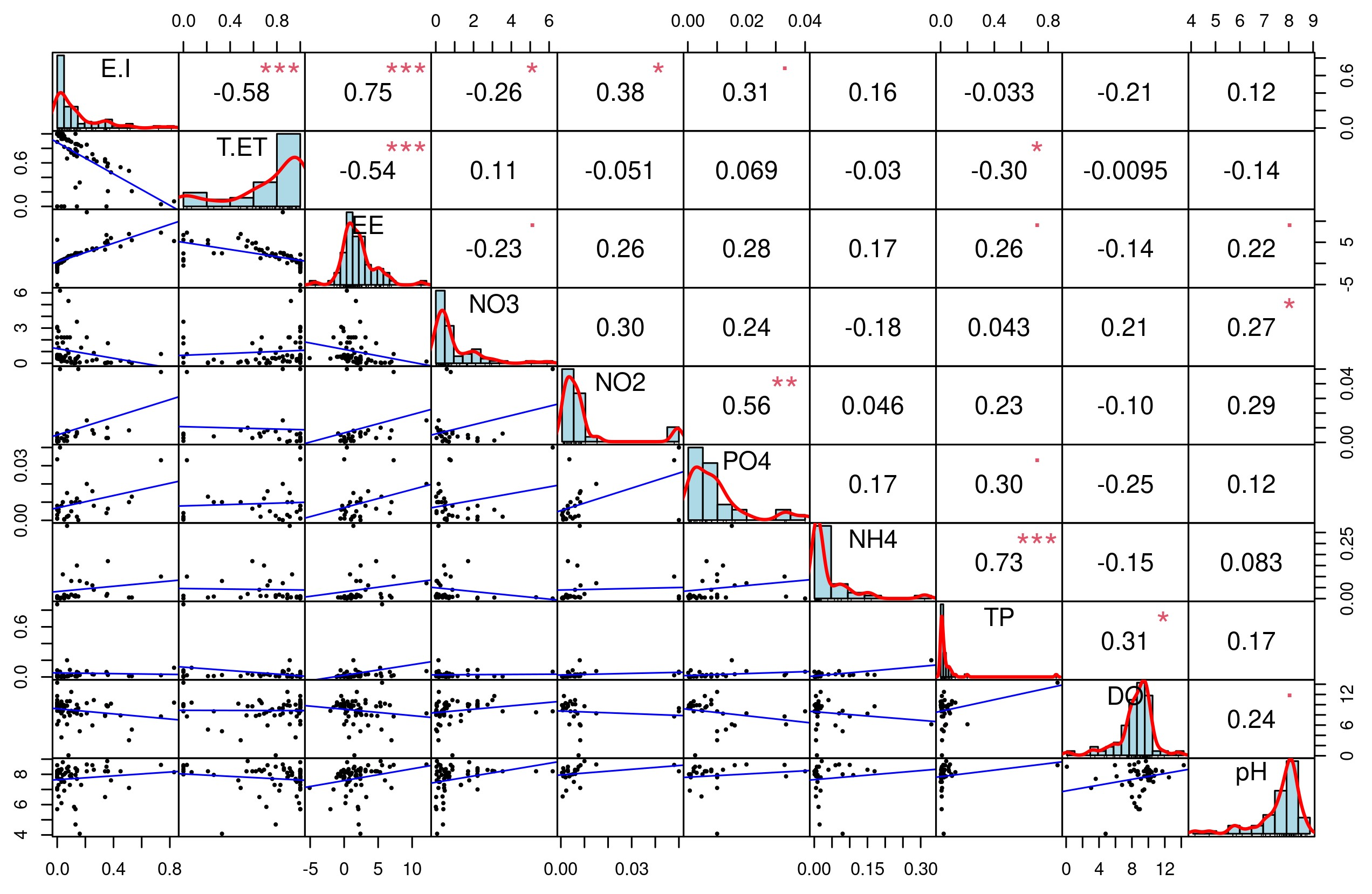


## **Figure S2. Correlation matrix between variables. The areas of the circles show the absolute value of the corresponding correlation coefficient. E/I: evaporation to inflow; T/ET: transpiration fluxes; EE: evaporative enrichment; NO_3_^-^: nitrate (mg l^-1^); NO_2_^-^: nitrite (mg l^-1^); PO_4_^3-^ : orthophosphate (mg l^-1^); NH_4_^+^: total ammonium (mg l^-1^); TP: total phosphorus (mg l^-1^); DO: dissolved oxygen (%); and pH. Each significance level is associated to a symbol: *p*-values 0.001 (***), 0.01 (**), 0.05 (*).**

1. Supplementary Methods

Water quality and stable isotopes analyses

The water quality characteristics selected in this study are commonly used for freshwater (lake or reservoir) monitoring. These characteristics are reflected in water as a key indicator of eutrophication^4,5^, as one of the consequences of freshwater warming. In *situ*, Secchi depth (m) and dissolved oxygen (DO; mg l^-1^) were measured with a standard Secchi disk and DO probes (YSI Pro Odo, Xylem Inc., 2011-2014), respectively. Electrical conductivity (Cond; µS cm^-1^) and pH were measured using laboratory conductometer LF2000 (WTW, Germany) and Glass pH electrodes (combined, Radiometer), respectively. Chlorophyll-a (Chl-a; µg l^-1^) concentration was determined spectrophotometrically after acetone extraction^6^. Total nitrogen (TN, mg l^-1^) was analysed on a Shimadzu TOC-LCPH analyser in unfiltered samples. Concentrations of total ammonium (NH_4_^+^; mg l^-1^), nitrate (NO_3_^-^; mg l^-1^) were determined by ion chromatography (Dionex IC25 and DIONEX 5000+, USA) and according to Kopacek and Prochazkova^7^. Nitrite (mg l^-1^) concentration was measured colorimetrically by a rubazoic acid method^7^. Total phosphorus (TP; mg l^-1^) and orthophosphate (PO_4_^3-^ ; mg l^-1^) concentrations were determined using molybdate method^8^.

The isotopic composition of the samples was analyzed using the Triple Liquid Water Isotope Analyser (from Los Gatos Research; T-LWIA) based on the principle of high-resolution laser absorption spectroscopy. This advanced analytical technique enables simultaneous measurements of *δ*^18^O, *δ*^2^H, and *δ*^17^O, providing a comprehensive isotopic fingerprint of the measured samples. Samples were measured and evaluated against prepared laboratory standards of known isotopic composition. The isotopic ratios of these laboratory standards were verified by measuring against international standards (VSMOW2, SLAP2) made by the International Atomic Energy Agency (IAEA). To check the quality control, the measurements of the samples were also interspersed with periodic measurements of the prepared verification samples with known isotopic composition. The final isotopic composition (*δ*^2^H and *δ*^18^O) was determined using LIMS software. Analytical error in case of *δ*^2^H was <1.0‰ and for *δ*^18^O was <0.25‰.

Data processing

The country-level map was generated from HydroBasin^2^ and processed in ArcGIS (version 10.6.1). Whereas, catchment areas were delineated using ArcGIS (version 10.6.1) with the hydrology function and a hydrologically conditioned raster DEM (3-arc-second spatial resolution; digital elevation model) from HydroSHEDS^9^. The delineated catchment boundaries were then converted from raster to a polygon to calculate the catchment area.

The original 23 land cover (LC) discrete classifications^10^ were reclassified into the following categories: bare land (areas with soil, sand, or rocks and <10% vegetation per year), cropland (dry and paddy fields), forest (encompassing coniferous and deciduous forests, herbaceous vegetation or wetlands, mosses, lichens, and shrubs), snow and ice, urban (comprising buildings and other manmade structures), and water (including lakes, reservoirs, and rivers).

For the climate type, we used the Köppen-Geiger classification data^1^. For annual average precipitation isotopes, we used the RCWIP data^11^, which utilize the regional precipitation-oxygen and -hydrogen data and empirically calculate the long-term monthly and annual isotopic composition at each location (1^o^ interval).

The NETCDF data (January 1, 2018 to March 31, 2023) from Copernicus^12^, after extraction by creating a multilayer raster object (i.e., RasterBrick), converting the time to a z-dimensional vector, and creating a data frame with the name of the layer and time using the packages “tidyverse”, “tibble” and “raster” from the R software version 3.6.3^11^, was used to calculate the average temperature, precipitation, and relative humidity of the catchment.

The Climate Engine^14,15^ database was used to obtain evapotranspiration (also known as actual evapotranspiration) data from January 2015 to December 2020. We used the TerraClimate raster (4 km, monthly) with average conditions. The groundwater table depth was obtained from HydroBasin^16^ and then calculated using the area percentage weighted distribution of the catchment.

Overall, after obtaining all individual lakes and/or reservoir catchments as shapefiles, we performed kriging and zonal statistics for the following raster input layers: climate type, precipitation isotopes, temperature, precipitation, relative humidity, and evapotranspiration.

Data treatment

All data analyses were performed using R software version 3.6.3^11^. Calculations and statistical tests were performed using without any transformation of data values. Normal distribution and correlation between variables were tested using the PerformanceAnalytics R package^11^. Principal component analysis (PCA) was calculated using the factoextra R package^17^. A conventional probability value (p-value) of 0.001, 0.01, and 0.05 were used to indicate significance for differences between variables^18^.

Isotope-enabled modeling

The regression line of Δ_L-P_*δ*^2^H ~ Δ_L-P_*δ*^18^O is referred to as the "local evaporation line" (LEL)^19^. The intersection point was defined as the area where the LEL crosses the Global Meteoric Water Line (GMWL)^17-19^. This intersection represents the estimated isotopic value of the lake's water input prior to evaporation. The universal GMWL as *δ*^2^H = 8 *δ*^18^O+10 was used due to the theoretical background^22^.

Runoff from the land surface and subsurface and direct precipitation into the lake influence the isotopic composition and can offset the effects of evaporation. To eliminate the influence of modern precipitation on the isotopic composition of the lake, we normalized our data of the mean isotopic composition of the lake (*δ*^18^O_L_ or *δ*^2^H_L_) to the isotopic composition of the volume-weighted precipitation in the catchment (*δ*^18^O_P_ or *δ*^2^H_P_) (Eq. 1):

*Δ_L-P_δ^18^O (Δ_L-P_δ^2^H) = δ^18^O_L_ (δ^2^H_L_) - δ^18^O_P_ (δ^2^H_P_)* (1)

The Δ_L-P_*δ*^18^O (Δ_L-P_*δ*^2^H) values are referred to as evaporative enrichment.

We determined the evaporation/input (E/I) ratios for a subset of 73 lakes. When the Isotope-enabled model showed a high discrepancy in the E/I ratio between the O and H two isotopes (i.e., >20% discrepancy), the lake was considered isotopically unbalanced^23^. Additionally, some lakes could not be considered because only δ^18^O was available for the water balance calculations, or the model could not be applied correctly assuming additional water inputs from deep groundwater recharge or glacier melts. We assumed hydrological steady-state conditions in the lake, which are mainly observed during the mixing period, and calculated the isotopic mass balance during this period^18-19^ as follows (Eq. 2):

*IδI = Q δQ + E δE* (2)

where *I* (m^3^ yr^-1^) is the total water inflow into the lake, including precipitation, surface, and subsurface flows; *E* is the evaporation flux; Q (m^3^ yr^-1^) is the lake outflow, and; *δ_I_* and *δ_Q_* (‰) are the amount-weighted (AW) mean isotopic compositions of the total inflow and outflow. The isotopic composition of the evaporation flux (*δ_E_*, ‰) was estimated using Eq 3^17, 22^:

$$\delta_{E}= \frac{\left( \frac{\delta_{E}- \varepsilon^{+}}{\alpha^{+}} \right)-h\delta_{A}-\varepsilon_{k}}{\left( 1-h+ \varepsilon_{k}{10}^{-3} \right)} (3)$$

where the isotopic composition of atmospheric moisture *δ_A_* was calculated based on an equilibrium approach^18-19^. The relative humidity (*h*), a dimensionless parameter, obtained from Copernicus NETCDF data ^12^. The isotopic separation equilibrium (*ε^+^*) was calculated based on the isotopic fractionation (*α^+^*)^25^. The kinetic isotopic separation (*ε_k_*) was calculated based on the relative humidity, transport resistant parameters (equal to one when the evaporation rate is controlled by molecular transport of water through the laminar layer to the atmosphere), and a kinetic constant^25^, similar to the approach described by Gibson *et al.*,^18-19^.

The evaporation to inflow ratio (E/I) of the lake was calculated according to by Gibson *et al.*, ^18-19^ Eq 4:

$$\frac{E}{I}= \frac{\left( \delta_{I}-\delta_{Q} \right)}{\left( \delta_{E}-\delta_{Q} \right)} (4)$$

Three lakes groups according to *E/I* value were determined: (i) ˂ 0.2 (lakes with low evaporation losses); (ii) 0.2 – 0.4 (lakes with moderate evaporation losses) and (iii) ˃ 0.4 (lakes with high evaporation losses)^23^.

The fraction of transpiration contribution in the evapotranspiration fluxes (T/ET, dimensionless value) was estimated based on a simple steady‐state representation of the catchment water balance similar to previous studies^18-19, 24, 26^ as Eq 5:

$$T/{ET= \frac{P-Q-E/{I.P}}{P-Q}} (5)$$

where *T* is transpiration, mm; *ET* is evapotranspiration, mm, which is composed of evaporation *E*, mm, and transpiration *T*, mm; *P* is precipitation, mm; *Q* is water yield, mm; and *E/I* is the isotope‐based evaporation loss from the watershed. The individual hydrological components were estimated using the methods described previously^18-19, 24, 26^. The T/ET categories were identified using Gaussian mixing model (GMM), a probabilistic model that assumes all the data points are generated from a mixture of a finite number of Gaussian distributions with unknown parameters. The expectation-minimization (EM) technique and Bayesian Information Criterion (BIC) were used to fit the combination in models and assess the data clusters, respectively^29^. According to T/ET value, the modelling findings identified three lake groups: (i) ˂ 0.3 (lakes with low *T/ET*); (ii) 0.3 – 0.6 (lakes with moderate *T/ET*) and (iii) ˃ 0.6 (*T/ET*).

Random Forest Model

Random Forest (RF), a supervised machine-learning algorithm, was chosen based on its proven applicability in hydrological, hydrogeological, and isotopic studies (e.g., Vystavna *et al.*,^21^). RF is a deep learning-based regression and classification tool, available in programming packages, and is being applied to an increasing amount of environmental data.

RF models were fitted using the “randomForest” package^11,30^. The RF targeted to predict the median E/I (primary variable) based on the set of climate and catchment variables (explanatory variables) (Table S1). The model was trained using a different number of trees (from 500 to 3000) and 2500 trees were selected for the final modelling. The selection was based on the stability of the out-of-bag (OOB) value and the general model performance (regression coefficient, R^2^). The physical meaningfulness of the explanatory variables in the RF model was tested using the mean decrease in accuracy (%IncMSE), which indicates how much the model accuracy decreases when the variables are eliminated and the mean decrease in Gini values (IncNodePurity), which is a measure of the importance of the variables based on the Gini impurity index used to calculate the splits in the trees. The higher the value of mean decrease accuracy or Mean Decrease Gini score, the more the importance of the variable to the model. Additionally, RF accuracy was tested using the variable importance measures based on the mean minimum depth calculated in one of the three ways specified by the parameter (mean_min_depth), total number of nodes that used for splitting (no_of_trees), total number of trees used for splitting the root node (times_a_root) and *p*-value for the one-sided binomial (no_of_trees vs. number of nodes) test. This test indicates whether the observed number of successes exceeds the theoretical number of successes if these were random (i.e., following the binomial distribution).

## **Table S1. Primary and the explanatory values (EV) used in the Random Forest model**

| **Factors used in the Random Forest** | | **Abbreviation** | **Units** | **Data source** |
| --- | --- | --- | --- | --- |
| Primary Factor | Evaporative enrichment | Δ_L-P_δ^18^O  (Δ_L-P_δ^2^H) | ‰ | Difference between lake and precipitation isotopes. Terzer-Wassmuth et al.^11^ provided precipitation weighted isotopic readings. |
| EV | Air temperature | T | ^o^C | Catchment weighted values were retrieved from Copernicus^12^ |
|  | Precipitation amount | P | mm yr^-1^ |  |
|  | Relative humidity | RH | % |  |
|  | Evapotranspiration | ET | mm yr^-1^ | Evapotranspiration calculated by Penman Monteith method taken Climate Engine^14,15^ |
| EV | Altitude | Alt | m as l | Obtained from different sources, when available from the studies. |
| EV | Lake depth | Depth | m |  |
| EV | Limnicity | Limn | 10x %Lake area in the catchment | Data obtained from HydroAtlas (2019) ^31^ |
| EV | Groundwater table depth | GWtab | cm | Data obtained from HydroAtlas (2019)^31^ with the reference on Fan et al.^16^ |
| EV | Bareland extent | Bare | % | Land covered with soil, sand, or rocks and <10% vegetation per year) |
| EV | Crop extent | Crop | % | Lands covered with temporary crops followed by harvest and a bare soil period (e.g., single and multiple cropping systems) and data obtained from 2019 discrete classification Copernicus Global Land Service^10^ |
| EV | Forest cover extent | Forest | % | Land areas with >10 % vegetation (coniferous and deciduous forests, herbaceous or wetlands, mosses, lichens, and shrubs) and the data obtained from 2019 discrete classification Copernicus Global Land Service^10^. |
| EV | Snow cover extent | Snow | % | Lands under snow or ice cover seasonally or throughout the year and derived from 2019 discrete classification Copernicus Global Land Service^10^ and and HydroAtlas map^31^ |
| EV | Urban extent | Urban | % | Land covered by buildings and other man-made structures and data derived from 2019 discrete classification Copernicus Global Land Service^10^ |
| EV | Catchment area | CatchArea | km^2^ | Derived from HydroSHEDS^9^ |
| EV | Surface area | Sarea | km^2^ | Derived from Google earth |

1. References

1. Kottek, M., Grieser, J., Beck, C., Rudolf, B. & Rubel, F. World Map of the Köppen-Geiger climate classification updated. *Meteorol. Zeitschrift* **15**, 259–263 (2006).

2. Lehner, B. & Grill, G. Global river hydrography and network routing: baseline data and new approaches to study the world’s large river systems. *Hydrol. Process.* **27**, 2171–2186 (2013).

3. Environmental Systems Research Institute (ESRI). ArcGIS Desktop 10.6.1. Redlands, CA: Environmental Systems Research Institute. (https://www.esri.com/). (2018).

4. Yu, G. *et al.* Recent advancement in water quality indicators for eutrophication in global freshwater lakes. *Environ. Res. Lett.* **18**, 063004 (2023).

5. Kakade, A. *et al.* World eutrophic pollution of lake and river: Biotreatment potential and future perspectives. *Environ. Technol. Innov.* **23**, 101604 (2021).

6. Lorenzen. Determination fo Chlorophyll and Pheophytin Pigments. *Plant Physiol.* 343–346 (1965).

7. Kopacek, J. & Prochazkova, L. Semi-Micro Determination of Ammonia in Water by the Rubazoic Acid Method. *Int. J. Environ. Anal. Chem.* **53**, 243–248 (1993).

8. Kopacek, J. & Hejzlar, J. Semi-Micro Determination of Total Phosphorus in Fresh Waters with Perchloric Acid Digestion. *Int. J. Environ. Anal. Chem.* **53**, 173–183 (1993).

9. Lehner, B., Verdin, K. & Jarvis, A. New Global Hydrography Derived From Spaceborne Elevation Data. *Eos, Trans. Am. Geophys. Union* **89**, 93–94 (2008).

10. Buchhorn, M. *et al.* Copernicus Global Land Service: Land Cover 100m: collection 3: epoch 2019: Globe (V3.0.1) [Data set]. *Zenodo* (2020) doi:10.5281/ZENODO.3939050.

11. Terzer-Wassmuth, S., Wassenaar, L. I., Welker, J. M. & Araguás-Araguás, L. J. Improved high-resolution global and regionalized isoscapes of δ18O, δ2H and d-excess in precipitation. *Hydrol. Process.* **35**, e14254 (2021).

12. Copernicus Climate Change Service, C. D. S. E-OBS daily gridded meteorological data for Europe from 1950 to present derived from in-situ observations. *Copernicus Climate Change Service (C3S) Climate Data Store (CDS)* https://cds.climate.copernicus.eu/cdsapp#!/dataset/10.24381/cds.151d3ec6?tab=overview (2020) doi:10.24381/cds.151d3ec6.

13. Team, R. D. C. A language and environment for statistical computing. R Foundation for Statistical Computing, Vienna, Austria. https://www.r-project.org/ (2021).

14. Huntington, J. L. *et al.* Climate Engine: Cloud Computing and Visualization of Climate and Remote Sensing Data for Advanced Natural Resource Monitoring and Process Understanding. *Bull. Am. Meteorol. Soc.* **98**, 2397–2410 (2017).

15. Climate Engine. Desert Research Institute and University of Idaho. Climate Engine. https://app.climateengine.org/climateEngine (2023).

16. Fan, Y., Li, H. & Miguez-Macho, G. Global patterns of groundwater table depth. *Science (80-. ).* **339**, 940–943 (2013).

17. Kassambara, A. & Mundt, F. factoextra: Extract and Visualize the Results of Multivariate Data Analyses. Package Version 1.0.7. *R Packag. version* **1**, (2020).

18. Davis, J. C. Analysis of Multivariate Data. *Stat. Data Anal. Geol. 3rd Ed.* 461 (2003).

19. Craig, H. & Gordon, L. I. Deuterium and oxygen 18 variations in the ocean and the marine atmosphere. 277–374 https://www.scienceopen.com/document?vid=3c68a140-4141-41c0-be2e-90f1e228e8a7 (1965).

20. Gibson, J. J., Birks, S. J., Yi, Y., Moncur, M. C. & McEachern, P. M. Stable isotope mass balance of fifty lakes in central Alberta: Assessing the role of water balance parameters in determining trophic status and lake level. *J. Hydrol. Reg. Stud.* **6**, 13–25 (2016).

21. Gibson, J. J. *et al.* Stable isotope mass balance of fifty lakes in central Alberta: Assessing the role of water balance parameters in determining trophic status and lake level. *JHyRS* **6**, 13–25 (2016).

22. Gat, J. R. Oxygen and hydrogen isotopes in the hydrological cycle. *Annu. Rev. Earth Planet. Sci.* **24**, 225–262 (1996).

23. Vystavna, Y., Harjung, A., Monteiro, L. R., Matiatos, I. & Wassenaar, L. I. Stable isotopes in global lakes integrate catchment and climatic controls on evaporation. *Nat. Commun. 2021 121* **12**, 1–7 (2021).

24. Gonfiantini, R. ENVIRONMENTAL ISOTOPES IN LAKE STUDIES. *Terr. Environ. B* 113–168 (1986) doi:10.1016/B978-0-444-42225-5.50008-5.

25. Horita, J. & Wesolowski, D. J. Liquid-vapor fractionation of oxygen and hydrogen isotopes of water from the freezing to the critical temperature. *Geochim. Cosmochim. Acta* **58**, 3425–3437 (1994).

26. Ferguson, P. R. & Veizer, J. Coupling of water and carbon fluxes via the terrestrial biosphere and its significance to the Earth’s climate system. *J. Geophys. Res. Atmos.* **112**, 24–30 (2007).

27. Gibson, J. J., Birks, S. J. & Yi, Y. Stable isotope mass balance of lakes: a contemporary perspective. *Quat. Sci. Rev.* **131**, 316–328 (2016).

28. Vystavna, Y. *et al.* Isotopic response of run-off to forest disturbance in small mountain catchments. *Hydrol. Process.* **32**, 3650–3661 (2018).

29. Reynolds, D. *Gaussian Mixture Models*. *Encyclopedia of Biometrics* (Springer, Boston, MA, 2009). doi:10.1007/978-0-387-73003-5_196.

30. Liaw, A. & Wiener, M. Classification and regression by randomForest. *R J.* **2**, 18–22 (2002).

31. Linke, S. *et al.* Global hydro-environmental sub-basin and river reach characteristics at high spatial resolution. *Sci. Data 2019 61* **6**, 1–15 (2019).
